# Transmembrane Tumor Necrosis Factor Controls Myeloid-Derived Suppressor Cell Activity *via* TNF Receptor 2 and Protects from Excessive Inflammation during BCG-Induced Pleurisy

**DOI:** 10.3389/fimmu.2017.00999

**Published:** 2017-08-25

**Authors:** Leslie Chavez-Galan, Dominique Vesin, Husnu Uysal, Guillaume Blaser, Mahdia Benkhoucha, Bernhard Ryffel, Valérie F. J. Quesniaux, Irene Garcia

**Affiliations:** ^1^Department of Pathology and Immunology, Centre Medical Universitaire (CMU), Faculty of Medicine, University of Geneva, Geneva, Switzerland; ^2^Laboratory of Integrative Immunology, National Institute of Respiratory Diseases “Ismael Cosio Villegas”, Mexico City, Mexico; ^3^CNRS, UMR7355, Orleans, France; ^4^Experimental and Molecular Immunology and Neurogenetics, University of Orléans, Orléans, France

**Keywords:** transmembrane tumor necrosis factor, TNF receptor 2, myeloid-derived suppressor cells, BCG infection, BCG-induced pleurisy

## Abstract

Pleural tuberculosis (TB) is a form of extra-pulmonary TB observed in patients infected with *Mycobacterium tuberculosis*. Accumulation of myeloid-derived suppressor cells (MDSC) has been observed in animal models of TB and in human patients but their role remains to be fully elucidated. In this study, we analyzed the role of transmembrane TNF (tmTNF) in the accumulation and function of MDSC in the pleural cavity during an acute mycobacterial infection. *Mycobacterium bovis* BCG-induced pleurisy was resolved in mice expressing tmTNF, but lethal in the absence of tumor necrosis factor. Pleural infection induced MDSC accumulation in the pleural cavity and functional MDSC required tmTNF to suppress T cells as did pleural wild-type MDSC. Interaction of MDSC expressing tmTNF with CD4 T cells bearing TNF receptor 2 (TNFR2), but not TNFR1, was required for MDSC suppressive activity on CD4 T cells. Expression of tmTNF attenuated Th1 cell-mediated inflammatory responses generated by the acute pleural mycobacterial infection in association with effective MDSC expressing tmTNF and interacting with CD4 T cells expressing TNFR2. In conclusion, this study provides new insights into the crucial role played by the tmTNF/TNFR2 pathway in MDSC suppressive activity required during acute pleural infection to attenuate excessive inflammation generated by the infection.

## Author Summary

Tumor necrosis factor (TNF) is an essential cytokine for host protection and control of tuberculosis (TB) infection that remains one of the leading causes of morbidity and mortality worldwide. Pleural TB is a frequent form of extra-pulmonary TB observed during a primary TB infection or after reactivation. Accumulation of myeloid-derived suppressor cells (MDSC) limiting T cell responses has been previously observed in TB patients. We have evaluated the role of TNF in MDSC function during acute infection in a murine model of BCG-induced pleurisy. We observed that transmembrane TNF (tmTNF) is crucial for the activity of MDSC and that tmTNF expressed on MDSC interacts with CD4 T cells expressing TNF receptor 2 (TNFR2) for suppressive activity that regulates the inflammatory process associated with pleural mycobacterial infection. This work highlights the essential role of tmTNF during acute mycobacterial pleurisy that is required to attenuate the excessive inflammatory response associated with pleural mycobacterial infection.

## Introduction

Tuberculosis (TB) is an infectious disease that remains a major health problem worldwide causing high morbidity and mortality. The pulmonary form is the most common form of TB infection but extra-pulmonary TB accounts for about one-third of reported TB cases (1). Generally, host immunity to a primary TB infection is able to mount an effective immune response inducing Th1-type cytokines, but in a minority of infected individuals, immunity appears inefficient resulting in an active TB (2). Pleural TB is considered as a form of extra-pulmonary TB which is a frequent clinical problem consisting in the accumulation of fluid and pleural cells in the pleural cavity subsequent to *Mycobacterium tuberculosis* infection (3, 4). Pleural TB has been reported as a primary TB pleurisy consequent to the rupture of pulmonary subpleural caseous lesions into the pleural space (5). Pleural TB can also be observed in patients with reactivation of latent TB and, in certain cases, associated with the use of corticosteroid and anti-TNF treatments or presence of comorbidities as HIV/AIDS and diabetes (6).

During acute pleural mycobacterial infection, the activity of inflammatory cells can be controlled by tolerogenic cells that attenuate the inflammatory process associated with the infection. Among these, MDSC are a heterogeneous population of innate cells that expand during cancer, inflammation, and infection, and play different roles depending on pathological processes (7). MDSC have been described as natural suppressor cells inhibiting the proliferative response of T-helper lymphocytes. MDSC have been distinguished as two distinct phenotypes: polymorphonuclear Ly6G^+^GR-1^high^ and mononuclear Ly6G^−^GR-1^dim^ MDSC (8). High frequencies of MDSC in blood and lung of patients with TB have been reported (9–11). In BCG vaccination studies in mice, MDSC were shown to restrain T cell priming by NO-dependent mechanisms (12). In a murine model of TB, MDSC have been shown to accumulate in the lung and other organs during progressive TB (13, 14). A study has reported that during chronic TB infection, there was excessive MDSC accumulation in the lung of sensitive mice and their depletion ameliorated disease outcome (15). The studies reported so far on MDSC activity during mycobacterial infection have been performed during chronic TB infection and results have shown that expansion of MDSC is associated with severity of the infection as MDSC prevent immune responses against mycobacteria (16).

Tumor necrosis factor is an important cytokine involved in the pathogenesis of several human inflammatory diseases and host defense mechanisms against many pathogens. TNF is first synthesized as a precursor or transmembrane form (tmTNF) and then cleaved by the TNF-α converting enzyme (TACE) under any stimuli which induce TNF producing soluble TNF (solTNF) (17). Using genetic mouse models expressing a mutated transmembrane form of TNF that cannot be cleaved by TACE (tmTNF KI mice), it has been shown that tmTNF mediates host protection against *Mycobacterium bovis* BCG and acute *M. tuberculosis* infections (18–21). We have also shown that inhibition of solTNF, by dominant-negative TNF biologics that do not block tmTNF, preserved immunity during acute BCG and *M. tuberculosis* infections and this treatment was efficient in preventing acute liver injury (22, 23). Anti-TNF therapies neutralizing soluble and tmTNF have shown their efficacy for the treatment of autoimmune inflammatory diseases; however, the mechanisms by which TNF can control immune tolerance during infection and how this can be disrupted by TNF inhibition remains unclear.

Recent studies on MDSC in the context of chronic inflammation have shown that TNF can block differentiation of MDSC and increase their intrinsic suppressive function (24). Inhibition of TNF during chronic inflammation decreased MDSC suppressive activity and enhanced maturation toward macrophages and dendritic cells restoring *in vivo* immune functions (24). More recently, using a model of sterile inflammation, it has been shown that membrane expression of TNFR2 on MDSC was required for differentiation and functionality (25). TNF signaling through TNFR2 promoted survival of MDSC helping tumor evasion (26). In mouse models of carcinogenesis, neutralization of TNF resulted in reduced MDSC accumulation and delayed the tumor growth (27). Together, these data show that the TNF pathway plays a critical role in the regulation of MDSC function. However, at present, whether TNF is required for MDSC accumulation and activity during acute mycobacterial infection and whether the TNF interaction with either TNFR1 or TNFR2 is required for MDSC suppressive function is unclear.

In this study, we have investigated the role of tmTNF in MDSC generation and suppressive activity in a model of acute pleural mycobacterial infection. Our data show that tmTNF on MDSC interact with CD4 T cells expressing TNFR2 but not TNFR1. TmTNF-TNFR2 interaction plays a critical role for MDSC suppressive activity on T cells which allows attenuation of the inflammation within the pleural cavity. Our data indicate that MDSC exert a beneficial function limiting inflammation during acute mycobacterial infection and favoring disease resolution.

## Materials and Methods

### Animals

C57BL/6 wild-type (WT), deficient for TNF (TNF KO) (28), and transmembrane form TNF knockin (tmTNF^Δ1–9,K11E^, deletion of amino acids 1–9 and substitution at position 11) (29) TNFR1 KO mice (30) and double TNFR1/TNFR2 (Jackson laboratory) (31) were used. CD4cre/TNFR2fl/fl mice that do not have TNFR2 on the surface of T cells were obtained by crossing C57BL/6NTac-Tg(CD4-cre) (32) (from Taconic farms) with TNFR2fl/fl mice (from EUCOMM *via* Institut Clinique de la souris, France from Prof Daniela Mannel, University of Regensburg, Germany). For experiments, adult mice (8–12 week old) were housed in animal facility of the Medical Faculty, University of Geneva (Geneva, Switzerland). All animal experiments were carried out in accordance with institutional guidelines and were approved by the academic ethical committee on animal experimentation and the cantonal veterinary office from Geneva (authorization No. GE167/14).

### *M. bovis* BCG

*Mycobacterium bovis* BCG Pasteur strain 1173 P2 was grown in Middlebrook 7H9 broth containing ADC (Difco), and middle-log phase bacilli were washed and frozen aliquots kept frozen at −80°C until use.

### *M. bovis* BCG Infection

BCG-induced pleurisy infection was generated by intrapleural cavity injection of 10^6^ CFU of *M. bovis*. BCG in 100 µL of saline as previously reported (33). Mice were monitored twice a week for body weight and sacrificed at day 14 post-infection or followed for survival studies. Groups of naïve littermates or uninfected mice were analyzed in the same way as infected animals.

### Pleural Cell and Fluid Preparation

Thoracic cavities from naïve and infected mice were washed twice with 1 mL of 2 mmol/L EDTA-phosphate-buffered saline (PBS), samples were centrifuged, and supernatants containing pleural fluid were frozen at −80°C for cytokine evaluation. Pleural cells were suspended in PBS-1% bovine serum albumin, counted, and used for the different techniques such as Flow cytometry, enrichment of MDSC, and cytospin followed by May-Grünwald-Giemsa and Ziehl-Neelsen (ZN) staining as reported (33).

### Multiparametric Flow Cytometry Analysis

The frequency of immunological cellular subpopulations in pleural cells was analyzed by flow cytometry. Briefly, cells were stained for 30 min at 4°C with different combinations of the following fluorochrome-conjugated mAb: GR-1 (Clone RB6-8C5), F4/80 (Clone BM8), Ly6C (Clone HK1.4), CD3 (Clone 145-2c11), and CD4 (Clone GK1.5) (BioLegend), and CD11c (Clone HL3) (BD Bioscience), and iNOS (Clone CXNFT) (Cell Signaling technology, eBioscience). After antibody incubation, cells were washed with PBA (phosphate buffered saline containing 0.1% Sodium Azide and 0.2% Albumin bovine). Data were collected using a FACs CyAn flow cytometer (Beckman Coulter, Inc.) and analyzed with FlowJo (Tree Star) software. 100,000 events were acquired per sample.

### Enrichment of MDSC

Single-cell suspensions were obtained from the pleural cavity of infected mice. MDSC were enriched by using magnetic microbeads kit (MDSC isolation kit; Miltenyi Biotec) and AutoMACS Pro Separator (Miltenyi Biotec). First, polymorphonuclear Ly6G^+^GR-1^high^ MDSC (PMN-MDSC) subpopulation were indirectly magnetically labeled with anti-Ly-6G and retained. The unlabeled cell fraction (depleted of Ly-6G^+^Gr-1^high^) was indirectly magnetically labeled with anti-GR1 and the mononuclear Ly6G^-^GR-1^dim^ MDSC (MO-MDSC) subpopulation isolated by positive selection. The purity of MDSC subpopulations was evaluated by flow cytometry (surface marker) and by cytospin (May-Grünwald-Giemsa stain) to identify morphology (PMN- vs MO- MDSC) and intracellular bacilli with ZN staining.

### *In Vitro* Proliferation and Suppression Assay of T Cells

Spleen cells from WT, TNFR1-KO, and TNFR2-CD4 KO mice were prepared as described previously (18). Bulk splenocytes were stimulated with plate-immobilized anti-CD3 (Clone 145-2C11) plus soluble anti-CD28 (Clone 37.51) (eBioscience), both antibodies were used at concentration of 1 µg/mL. Splenocytes were co-incubated with varying ratios of MDSC (1:1, 1:2, 1:4), after 48 h of co-culture at 37°C, supernatants were collected for cytokine measurements and cells for proliferation assay using KI-67 (Clone 16A8) (Biolegend). Briefly, cells were harvested, washed with PBA, and then surface molecules (CD3, CD4) were stained as described above. Subsequently, cell pellet was suspended in Fixation/permeabilization solution (eBioscience) at 4°C, washed with permeabilization buffer (eBioscience), and then stained with KI-67 for 30 min at 4°C. Cells were washed and analyzed by flow cytometry. Splenocytes with polyclonal stimuli were considered as 100% proliferation (Positive control).

### PCR Analysis for Genotyping CD4-cre TNFR2 Mice

DNA extracted from the tail was used for PCR to detect homozygous mice with TNFR2 deletion. Primers were CD4-cre F: 5′-CCCAACCAACAAGAGCTC-3′, and CD4-cre R: 5′-CCCAGAAATGCCAGATTAGG-3′. Amplifications were performed using the following program: preheating stage at 95°C for 3 min, 35 cycles at 95°C for 45 s, 56,7°C for 30 s, 72°Cfor 45 s, and extension at 72°C for 5 min.

### Flow-Sorting of CD4 T Cells from the Spleen

Spleen from 14-day infected mice was dissociated and CD4 T cells flow-sorted using magnetic microbeads kit (mouse CD4 T cells isolation kit; Miltenyi Biotec) and AutoMACS Pro Separator (Miltenyi Biotec).

### Cytokine Evaluation Enzyme-Linked Immunosorbent Assay (ELISA)

Cytokine levels in the pleural fluid and cell supernatants were assessed by ELISA. IL-2, IFN-α, IFN-γ, IL-12p70, IL-6, IL-10, and the chemokine CCL2 (MCP-1) were quantified in the pleura fluid and in cellular supernatants in accordance with the manufacturer’s instructions.

### Western Blot

Enriched MO-MDSC and PMN-MDSC were lysed and subjected to SDS-PAGE and transferred to membranes. The primary antibodies used were polyclonal Arginase 1 and iNOS (Cell Signaling Tech) and secondary antibody was horseradish peroxidase-conjugated goat anti-rabbit. Blots were developed with chemiluminescence substrate ECL. The density of the bands was analyzed using the online ImageJ 1.39c software. Actin was used as loading control as reported (34).

### Statistical Analysis

Statistical analyses were performed with GraphPad Prism software (GraphPad Soft., La Jolla, CA, USA). *P*-value <0.05 was considered as statistically significant. Experiments with two groups were analyzed with an unpaired Student’s *t*-test and one-way ANOVA followed by Bonferroni *post hoc* test for multiple comparisons.

## Results

### Transmembrane TNF is Sufficient to Rescue Mice from BCG-Induced Pleurisy and to Prevent Excessive Accumulation of Neutrophils in the Pleural Cavity

**Figure 1:**
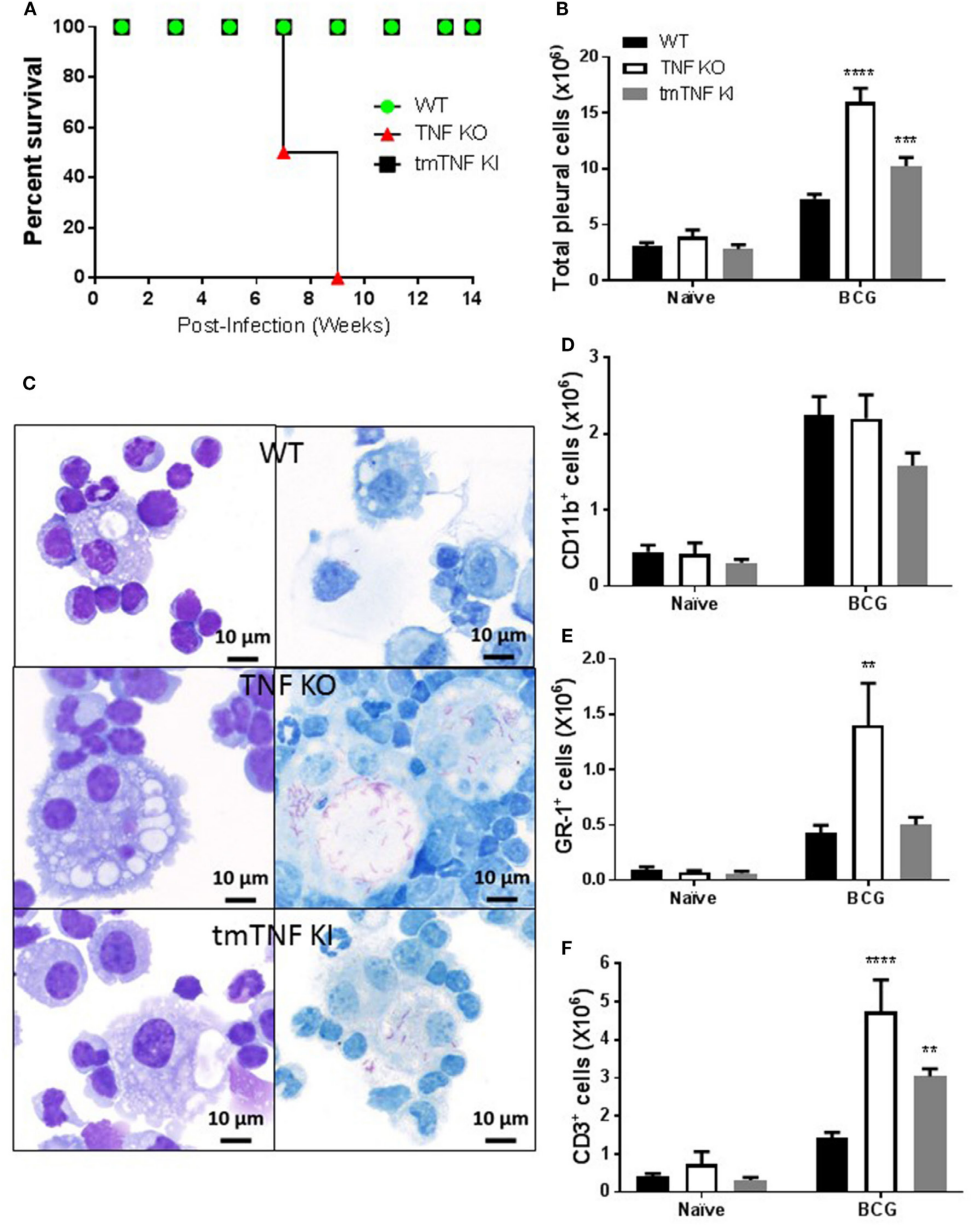
Membrane bound tumor necrosis factor (TNF) is sufficient to activate an efficient immune response during BCG-induced pleurisy. **(A)** The survival of BCG-infected mice was monitored for 14 weeks post-infection (*n* = 7–9 from two experiments). **(B)** Total number of cells from the pleural cavity recovered in naïve littermate or uninfected and in mice infected with BCG for 14 days. (Bar graphs show means ± SEM of four experiments (*n* = 6, naïve and *n* = 20, infected mice/per group) wild-type (WT) and TNF KO and infected tmTNF KI mice). **(C)** Photomicrographs representative from cytospin preparation with cells isolated from the pleural cavity at day 14 post-infection and stained with May-Grunwald-Giemsa (MGG) (left) and Ziehl-Neelsen (ZN) staining (right) that shows intracellular bacilli (red) in macrophages in WT and tmTNF KI and in giant cells in TNF KO cells. **(D)** Quantification of the percentage of CD11b^+^, **(E)** GR-1^+^, and **(F)** CD3^+^ cells was performed by flow cytometry analysis and absolute numbers were obtained considering the total cell number recovered from the pleural cavity in individual mouse (*n* = 4, naïve and *n* = 9, infected mice). Bar graphs show means ± SEM **(D–F)** of two experiments (**P < 0.001, ***P < 0.0001, and ****P < 0.00001 vs WT, ANOVA and Bonferroni *post hoc* test). Scale bars = 10 μm.

We have previously reported that a mouse model of pleural infection shows that TNF or its receptors are crucial to control *M. bovis* BCG-induced pleurisy (33). To further evaluate whether transmembrane TNF (tmTNF) or soluble-TNF form is required for protection during pleural mycobacterial infection, we analyzed mice that express a mutated form of TNF (tmTNF^Δ1–9,K11E^ or tmTNF KI) that cannot be cleaved by TACE and do not produce solTNF (29). As previously reported after an i.v. BCG infection, we observed that tmTNF KI mice were able to control BCG-induced pleurisy as they survived for more than 14 weeks post-infection as is the case for WT mice (Figure 1A). By contrast, TNF KO mice did not resist pleural BCG infection and died at 7–9 weeks after infection (Figure 1A). Pleural cavity cytokine profiles in WT and tmTNF KI mice exhibited no differences at 14 weeks post-infection, suggesting that tmTNF KI resolved the infection as observed in WT mice (Figure S1 in Supplementary Material). Evaluation of cells accumulated in the pleural cavity at day 14 post-infection showed higher cell numbers in tmTNF KI mice than in WT mice but TNF KO mice had twofold higher cell numbers compared with WT mice (Figure 1B). Pleural BCG infection in TNF KO, but not tmTNF KI mice resulted in expansion of multinucleated giant cells containing numerous vacuoles and many bacilli (Figure 1C). TNF KO cells were previously shown to be deficient in iNOS expression and unable to eliminate bacteria which led to a miliary TB (33). Accumulated cells were mainly myeloid CD11b^+^ cells and the total number was not affected by the absence of TNF (Figure 1D). Nevertheless, the number of GR1^+^ cells significantly increased in TNF KO but not in tmTNF KI mice suggesting that tmTNF controls neutrophil recruitment (Figure 1E). However, both solTNF and tmTNF regulated the recruitment of CD3 lymphocytes in BCG-infected mouse pleural cavity (Figure 1F). Our data show that tmTNF is mandatory for the control of cell recruitment and protection against BCG-induced pleurisy in mice.

### BCG-Induced Pleurisy Activates Expansion of Monocytic and Granulocytic MDSC

**Figure 2:**
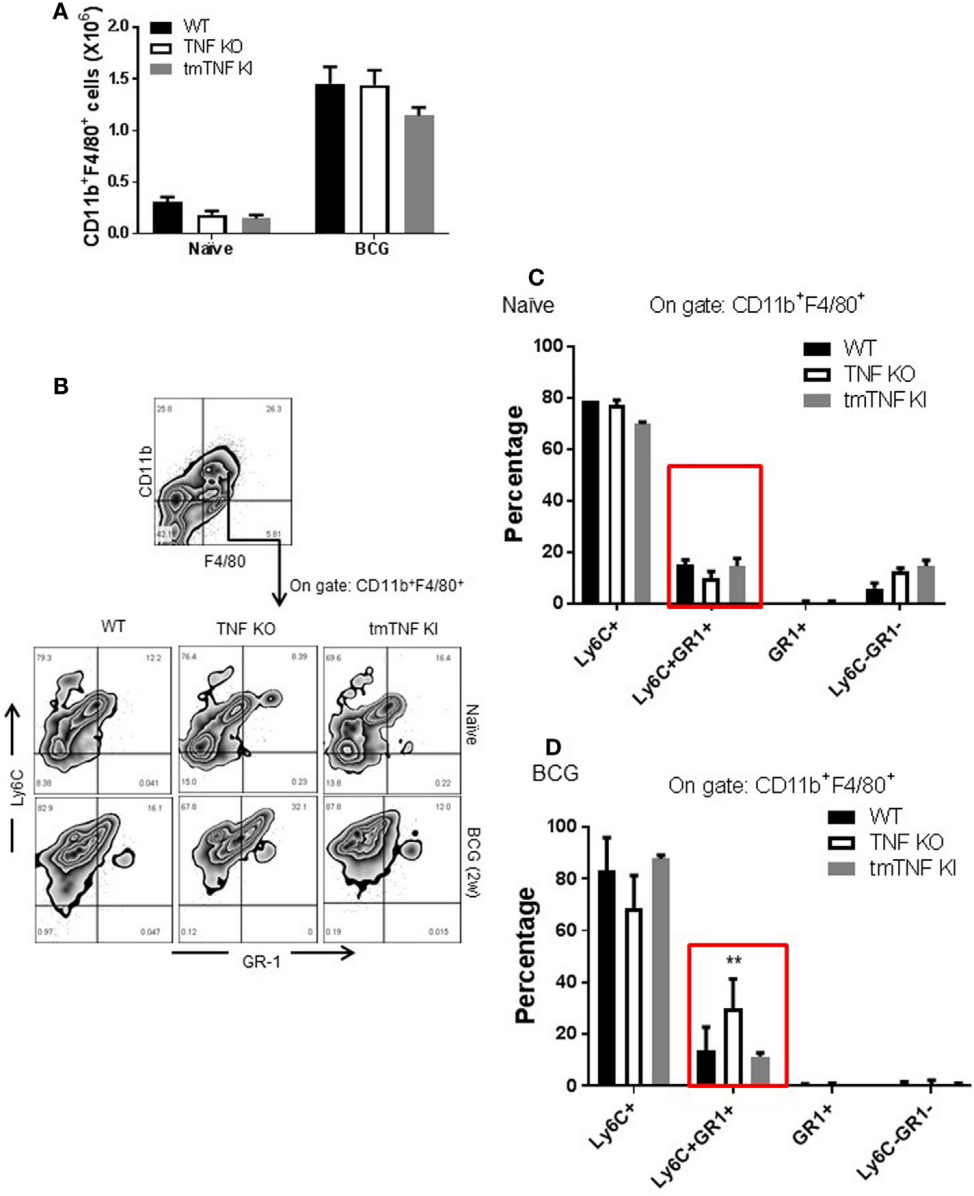
Absence of tumor necrosis factor (TNF) induces an elevated expansion of cells with a phenotype of myeloid-derived suppressor cells (MDSC) phenotype in the pleural cavity of BCG-infected mice which is restored by tmTNF. **(A)** Absolute numbers of CD11b^+^ F4/80^+^ cells obtained by flow cytometry analysis considering the total cell number per pleural cavity in individual mouse. **(B)** Representative zebra plot corresponding to the strategy used to identify Ly6C and GR1 coexpression on gated CD11b^+^ F4/80^+^ subsets. **(C)** The frequency of Ly6C^+^, Ly6C^+^GR1^+^, GR1^+^, and Ly6C^−^GR1^−^ subpopulations on gated CD11b^+^ F4/80^+^ cells is shown in naïve mice, and **(D)** in BCG- infected mice at day 14 post-infection. Bar graphs show means ± SEM of two experiments (*n* = 5, naïve, *n* = 9 infected mice wild-type (WT) and TNF KO, and *n* = 5 tmTNF KI mice, **P < 0.001 vs WT. ANOVA and Bonferroni *post hoc* test).

*Mycobacterium tuberculosis* infection in human and in mouse has been associated with the accumulation and expansion of MDSC which may contribute to aggravation and impaired control during chronic infection. We evaluated the presence of MDSC in the pleural cavity following BCG-induced pleurisy. Two types of analyses have been performed according to previous publications on MDSC during mycobacterial infection (13, 15). A gate on CD11b^+^ F4/80^+^ myeloid cell population showed that naïve TNF KO and tmTNF KI mice presented similar cell numbers compared to WT mice, but BCG infection induced a 6-fold increase at day 14 post-infection in all groups of mice (Figure 2A). Co-expression of Ly6C^+^ and GR1^+^ was evaluated on CD11b^+^ F4/80^+^ subset which may contain cells with a phenotype of MDSC (Figure 2B). The CD11b^+^ F4/80^+^ Ly6C^+^ GR1^+^ subpopulation expanded during infection and an increased frequency was found in TNF KO mice, but not in tmTNF KI mice, suggesting that tmTNF, but not solTNF regulates their expansion in the pleural cavity of infected mice (Figures 2C,D). To further confirm the expansion of cells with a phenotype of MDSC, a second analysis of pleural cells was done by evaluating cells co-expressing CD11b^+^ GR1^+^ as previously described (14). The analysis confirmed that this subset expanded during the infection and its frequency was higher in TNF KO mice compared with WT and tmTNF KI mice (Figure S2 in Supplementary Material).

**Figure 3:**
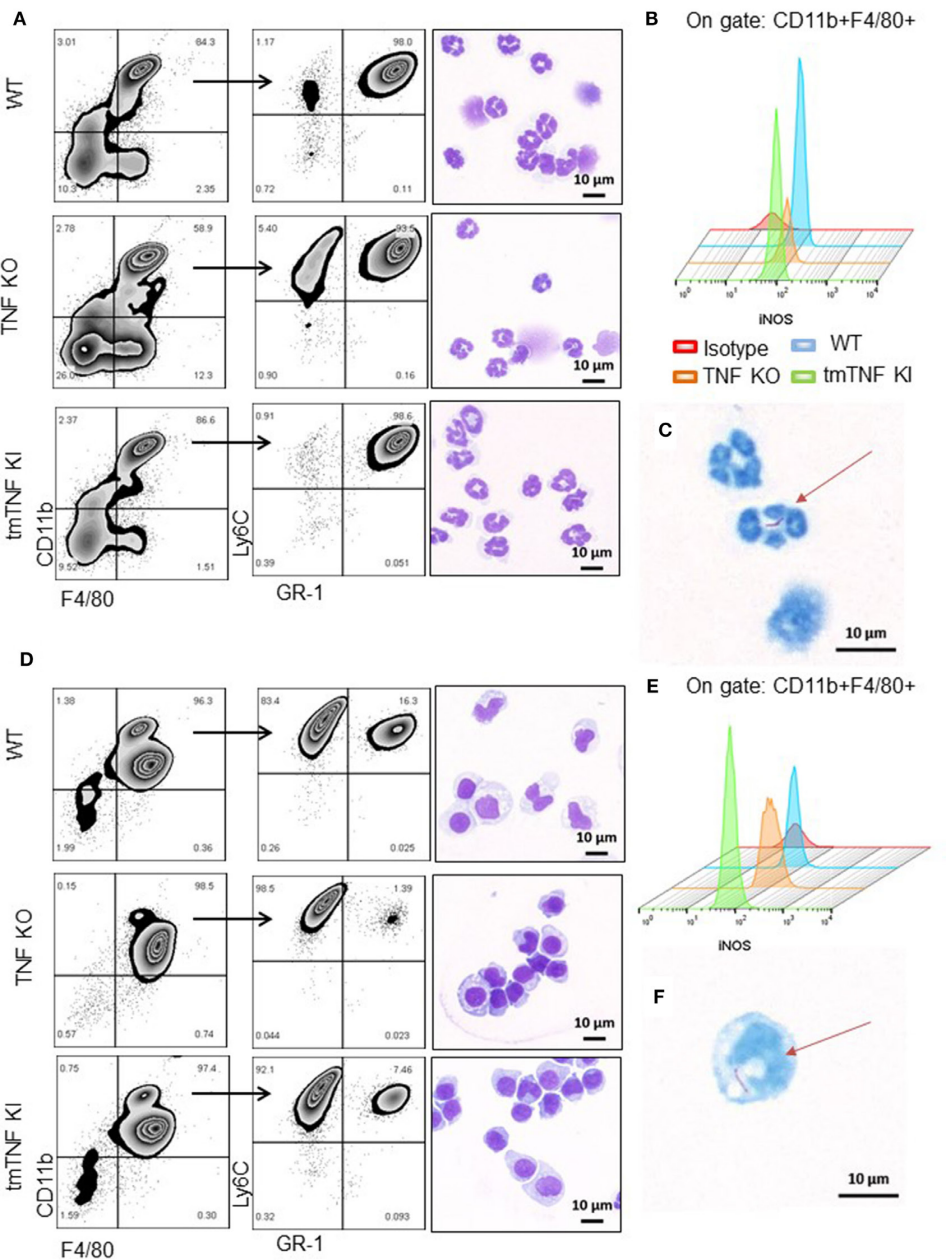
Presence of tmTNF restores the frequency of myeloid-derived suppressor cells and iNOS expression. MDSC from a pool of pleural cells (*n* = 5–7 mice per group) were flow-sorted using a MDSC kit. **(A)** Representative zebra plot corresponding to the analysis used to evaluate the purity of sorted PMN-MDSC by flow cytometry and morphology after staining with May-Grunwald-Giemsa (MGG). **(B)** Representative Stagger Offset histogram depicting the frequency of iNOS^+^ cells inside the gate of CD11b^+^ F4/80^+^ cells and comparison between wild-type (WT) (blue), tumor necrosis factor (TNF) KO (orange), and tmTNF KI (green) pleural MDSC. **(C)** Flow-sorted PMN-MDSC from TNF KO mice stained with Ziehl-Neelsen (ZN) and illustration of the presence of one BCG in the cell indicated by the arrow. **(D)** Representative zebra plot corresponding to the analysis used to evaluate the purity of MO-MDSC by flow cytometry and morphology after MGG stain. **(E)** Representative Stagger Offset histogram showing the frequency of iNOS^+^ cells inside the gate of CD11b^+^ F4/80^+^ pleural MDSC and comparing WT (blue), TNF KO (orange) and tmTNF KI (green) cells. **(F)**. Flow-sorted MO-MDSC from TNF KO mice stained with ZN and illustration of the presence of one BCG in the cell indicated by the arrow. Data are representative of two experiments.

To explore if these subpopulations contain functional MDSC, pleural cells at day 14 post-infection were fractionated into two subpopulations according to the presence of GR1 using MDSC-isolation kits as described (13). A first fraction was defined as granulocytic or polymorphonuclear MDSC cells (PMN-MDSC) and characterized by flow cytometry as CD11b^+^F4/80^+^Ly6G^+^GR1^high^Ly6C^int^ (Figure 3A). Further examination by light microscopy confirmed a polymorphonuclear phenotype as expected (Figure 3A). Flow cytometry analysis of iNOS expression showed that tmTNF was sufficient to maintain the expression of iNOS in the MDSC population; however, a small fraction of PMN-MDSC from TNF KO produced iNOS (Figures 3A,B). The second isolated fraction was characterized as CD11b^+^F4/80^+^Ly6G^−^GR1^dim^Ly6C^high^ and the phenotype defined as mononuclear MDSC cells (MO-MDSC). WT and tmTNF KI MO-MDSC showed iNOS expression that was lower for TNF KO cells (Figures 3D,E). The poor ability of TNF KO MDSC to produce iNOS was confirmed using CD11b^+^ GR1^+^ as main MDSC markers after flow-sorting (Figures S3A,B in Supplementary Material). The capacity of MDSC to contain intracellular mycobacteria was evaluated. Analyses of cells containing bacilli by ZN staining of MDSC sorted preparations revealed that phagocytic MDSC is a very rare event for pleural MDSC. We could observe few PMN-MDSC and MO-MDSC containing one or two bacilli in TNF KO cells (Figures 3C,F). The frequency of MDSC containing bacilli in WT and tmTNF KI cells was very low. Our data show that BCG-induced pleurisy induces accumulation of MDSC in the pleural cavity and tmTNF regulates their accumulation.

### Transmembrane TNF Restores Mononuclear MO-MDSC and Polymorphonuclear PMN-MDSC Suppressive Functions on CD4 T Cells

**Figure 4:**
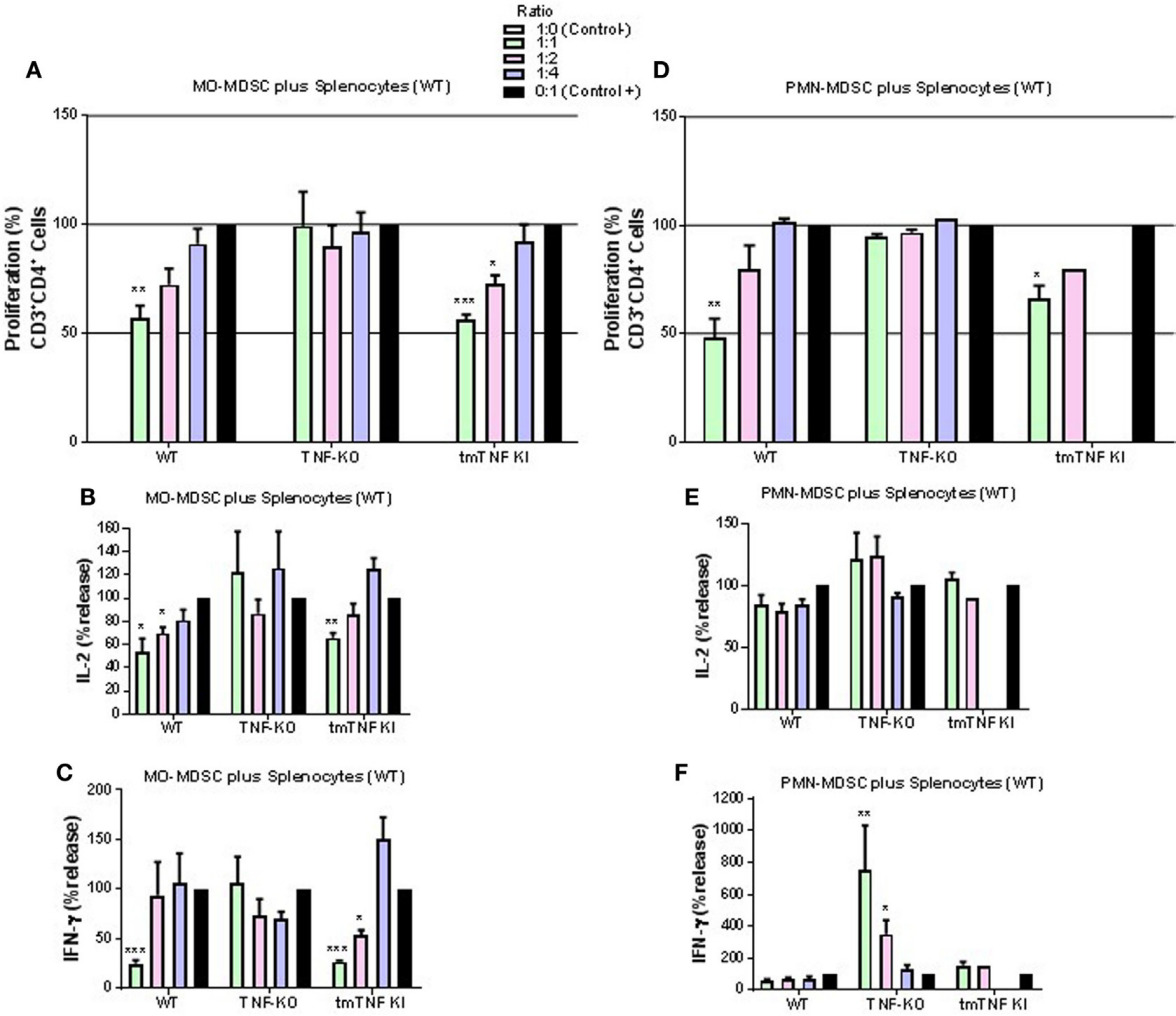
Expression of tmTNF restores myeloid-derived suppressor cells (MDSC) suppressive function on CD4 T cells. **(A)**. Proliferation of CD3 CD4 T cells after polyclonal stimulation and in the presence or absence of flow-sorted pleural mononuclear MO-MDSC (ratio MDSC:Splenocytes, 1:1, 1:2, and 1:4) was measured by flow cytometry using KI-67 after 48 h of co-culture. Pools of pleural cells were from 5 to 7 mice per group. **(B)** IL-2 and **(C)** IFN-γ production from supernatants of splenocytes and MO-MDSC co-cultures at different ratio. **(D)** Proliferation of CD3 CD4 T cells after polyclonal stimulation and in the presence or absence of flow-sorted pleural polymorphonuclear PMN-MDSC co-cultured with splenocytes for 48 h. **(E)** IL-2 and **(F)** IFN-γ production from co-cultures of PMN-MDSC and splenocytes. MDSC alone were used as the negative control and activated splenocytes as positive controls (100%). Bar graphs show means ± SEM. Data are representative of three independent experiments (*n* = 3–6 per group, *P < 0.05, **P < 0.001, and ***P < 0.0001 vs positive control. ANOVA and Bonferroni *post hoc* test).

Considering the phenotypic similarity of the two defined myeloid fractions with reported MDSC, we assessed their functional characteristic in terms of suppression of T cell proliferation by flow cytometry (Figure S4 in Supplementary Material). Co-culture experiments of pleural MO-MDSC (Ly6G^−^GR-1^dim^) from WT mice with stimulated splenocytes from naïve mice revealed a partial inhibition of CD4 T cell proliferation in a dose-dependent manner (Figure 4A). By contrast, MO-MDSC from TNF KO were not able to reduce CD4 T cell proliferation, while MDSC from tmTNF KI inhibited CD4 T cell proliferation similar to WT cells (Figure 4A). In addition, co-cultures of both WT and tmTNF KI MO-MDSC and splenocytes reduced IL-2 and IFN-γ production but not of TNF KO MO-MDSC (Figures 4B,C). Pleural PMN-MDSC (Ly6G^+^GR1^high^) from WT mice inhibited CD4 T cell proliferation in a dose-dependent manner (Figure 4D). PMN-MDSC from TNF-KO did not inhibit CD4 T cell proliferation, whereas tmTNF KI PMN-MDSC inhibited CD4 T cell proliferation (Figure 4D). However, PMN-MDSC co-cultures did not suppress IL-2 and IFN-γ responses as is the case for MO-MDSC, but surprisingly, TNF KO MDSC activated IFN-γ production with an 80-fold increase in a dose-dependent manner (Figures 4E,F). Together, these data show that tmTNF mediates the suppressive function of MDSC on CD4 T cells.

### Interactions of MDSC Expressing tmTNF with TNFR2 on CD4 T Cells Is Required for MDSC Suppressive Function

**Figure 5:**
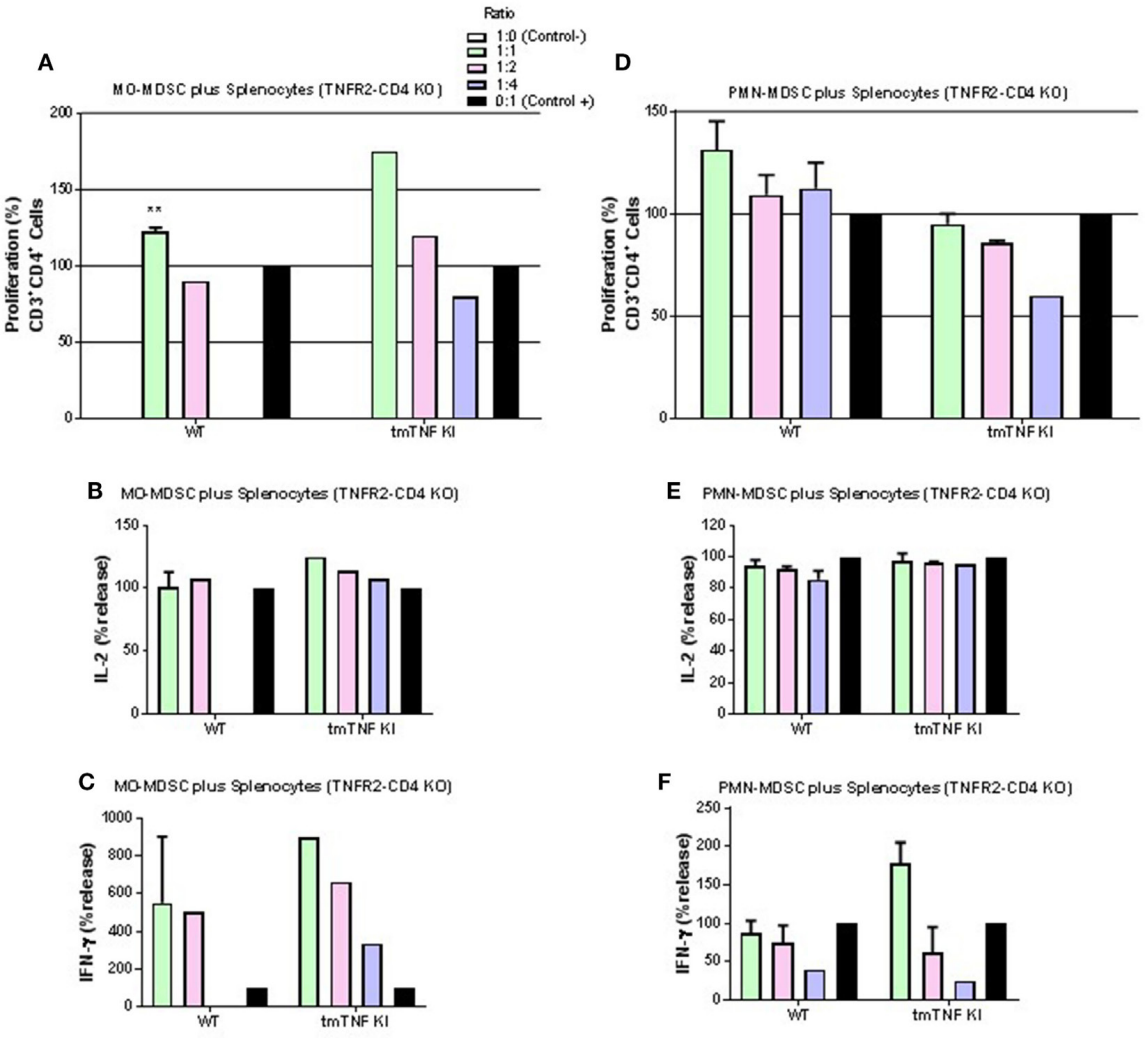
Interaction of tmTNF and TNF receptor 2 (TNFR2) is required for suppressive function of myeloid-derived suppressor cells (MDSC) on CD4 T responses. **(A)** Proliferation of CD3 CD4 T cells without TNFR2 (TNFR2-CD4 KO) after polyclonal stimulation and in the presence or absence of flow-sorted pleural mononuclear MO-MDSC (ratio MDSC:Splenocytes, 1:1, 1:2 and 1:4) was measured by flow cytometry using KI-67 after 48 h co-culture. **(B)** IL-2 and **(C)** IFN-γ production from splenocyte and MO-MDSC co-cultures at different ratios. **(D)** Proliferation of CD3 CD4 T cells without TNFR2 (TNFR2-CD4 KO) after polyclonal stimulation and in the presence or absence of flow-sorted pleural polymorphonuclear PMN-MDSC co-cultured with splenocytes for during 48 h. **(E)** Il-2 and **(F)** IFN-γ production from co-cultures of PMN-MDSC and splenocytes. MDSC alone were used as negative control and activated splenocytes as positive controls. (Bar graphs show means ± SEM of *n* = 4–6 from two independent experiments, **P < 0.001 vs positive control. ANOVA and Bonferroni *post hoc* test).

We further asked if a specific TNFR is required for the CD4 T cell interaction with MDSC expressing tmTNF. Pleural MDSC cells from BCG-infected mice were co-cultured with activated splenocytes from mice whose CD4 T cells do not express TNFR2 (TNFR2-CD4 KO). We observed that MO-MDSC from either WT or tmTNF KI mice did not exhibit any suppressive activity on activated CD4 T cells and surprisingly, lymphocytes appeared to proliferate with increasing amounts of MO-MDSC (Figure 5A). The levels of IL-2 were not changed and the level of IFN-γ increased in a dose-dependent from MO-MDSC (Figures 5B,C). Similarly, PMN-MDSC from WT mice or tmTNF KI did not suppress CD4 T cell proliferation, but rather PMN-MDSC increased the frequency of CD4 T cell proliferation (Figure 5D). The levels of IL-2 were not affected and IFN-γ levels increased with increasing amounts of PMN-MDSC (Figures 5E,F).

**Figure 6:**
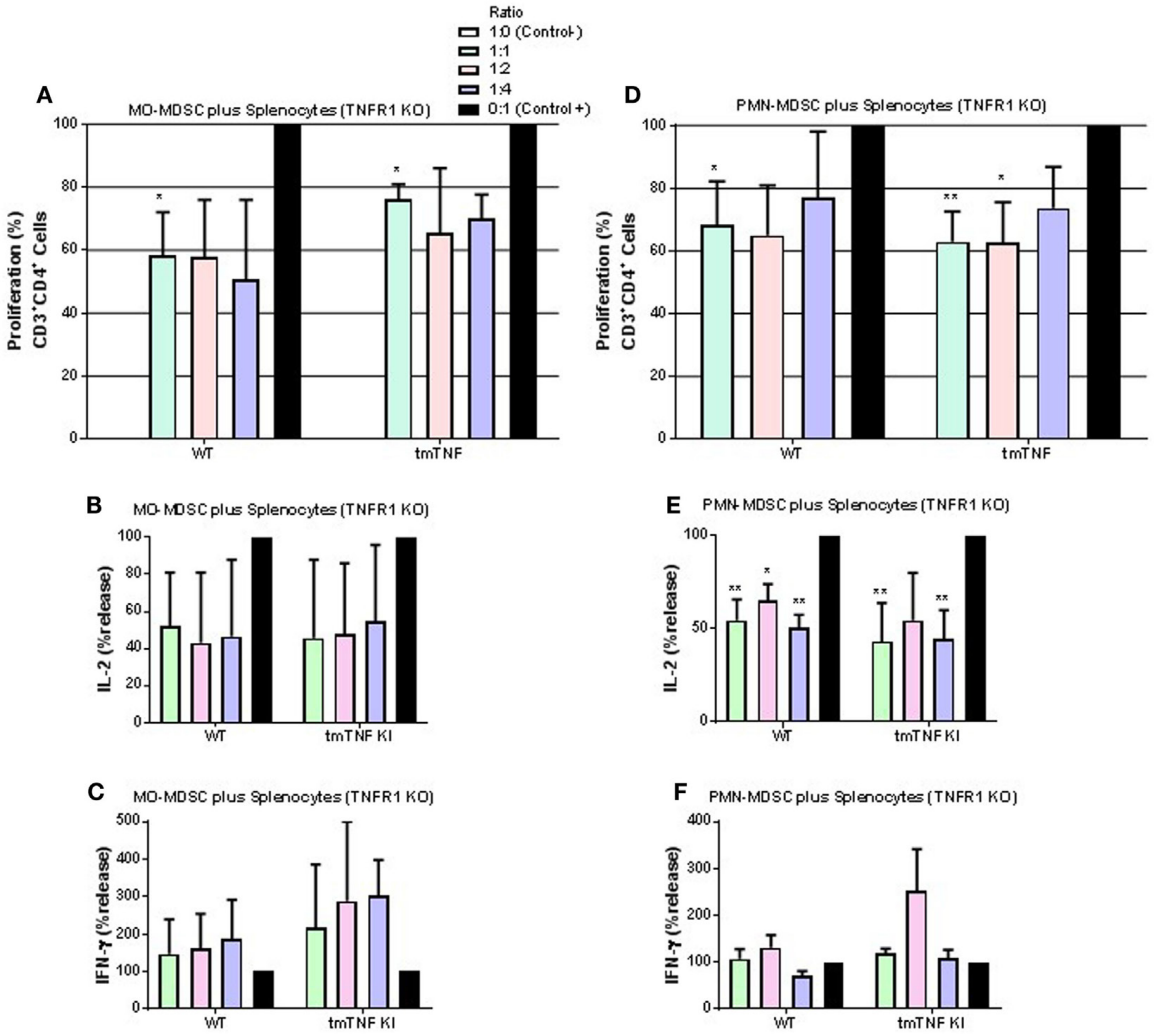
TNFR1 is not necessary for suppressive function of myeloid-derived suppressor cells (MDSC) on CD4 T cell responses. **(A)** Proliferation of CD3 CD4 T cells from TNFR1 KO mice after polyclonal stimulation and in the presence or absence of flow-sorted pleural mononuclear MO-MDSC (ratio MDSC:Splenocytes, 1:1, 1:2, and 1:4) was measured by flow cytometry using KI-67 after 48 h of co-cultures. **(B)** IL-2 and **(C)** IFN-γ production from supernatants of splenocytes and MO-MDSC co-cultures at different ratio. **(D)** Proliferation of CD3 CD4 T cells from TNF KO mice after polyclonal stimulation and in the presence or absence of flow-sorted pleural polymorphonuclear PMN-MDSC co-cultured with splenocytes for 48 h. **(E)** IL-2 and **(F)** IFN-γ production from co-cultures of PMN-MDSC and splenocytes. MDSC alone were used as the negative control and activated splenocytes as positive controls (100%). (Bar graphs show means ± SEM of *n* = 5–7 mice per group from two independent experiments, *P < 0.05 and **P < 0.001 vs positive control. ANOVA and Bonferroni *post hoc* test).

We then tested whether absence of TNFR1 on CD4 T cells would affect responses to MDSC. MO-MDSC from WT and tmTNF KI mice induced suppression on T cell proliferation and on IL-2 effects but not on IFN-γ in the absence of TNFR1 on CD4 T cells (Figures 6A–C). PMN-MDSC also showed suppressive activity on TNFR1 KO T cell proliferation and on IL-2 but not on IFN-γ production (Figures 6D–F). These data suggest that TNFR1 expression on CD4 T cells is not essential for interaction between MDSC and CD4 T cells to exert suppressive function. Previous reports have shown that TNF signaling drives MDSC accumulation and favors tumor cell evasion. To examine if TNF signaling was also important for MDSC activity during acute BCG infection, we used sorted pleural MDSC from BCG-infected TNFR1/TNFR2 KO mice. These mice were shown to be highly sensitive to both systemic and pleural BCG infection (31, 33). Both MO- and PMN-MDSC displayed complete absence of suppressive activity on CD4 T cells and even an enhancement of the proliferation of CD4 T cells co-cultured with MO-MDSC was observed (Figure S5 in Supplementary Material). Our results indicate that TNFR2 expression on lymphocytes is essential for the interaction with tmTNF to drive MDSC effector functions. In addition, absence of TNFRs on MDSC not only abolishes suppressive activity of MDSC but also activates proliferation and IFN-γ production of CD4 T cells.

### Transmembrane TNF Down-Regulates Excessive Inflammation during Acute BCG-Induced Pleurisy

**Figure 7:**
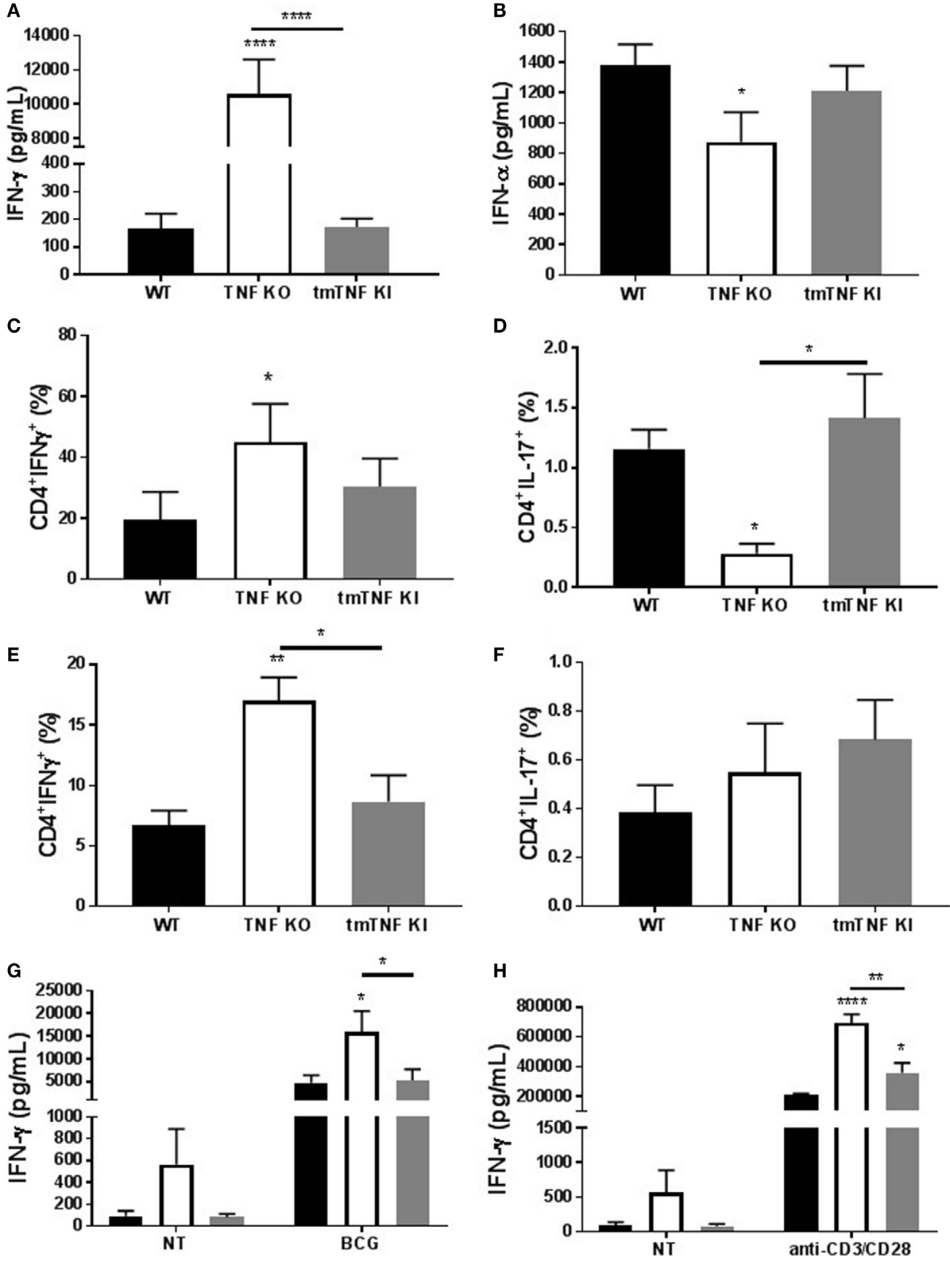
Transmembrane tumor necrosis factor (TNF) controls the excessive inflammatory response mediated by CD4 T cells. **(A)**, IFN-γ and **(B)** IFN-α levels in the pleural fluid of mice infected with BCG for 14 days (*n* = 9–15 mice per group). **(C)** The frequency of CD4 T cells producing IFN-γ or **(D)** IL-17 was assessed in pleura cells by flow cytometry. **(E)** The frequency of CD4 T cells producing IFN-γ or **(F)** IL-17 was assessed in splenocytes from mice infected with BCG. **(G)** IFN-γ levels from flow-sorted CD4 T cells from BCG-infected mice and cultured for 24 h with or without BCG at MOI 1. **(H)** IFN-γ levels from flow-sorted CD4 T cells from BCG-infected mice and cultured for 36 h with or without anti-CD3/CD28 beads. Bar graphs show means ± SEM (*n* = 6–8/per group, *P < 0.05, **P < 0.001, ****P < 0.00001 vs wild-type (WT), ANOVA and Bonferroni *post hoc* test).

We have reported that BCG-induced pleurisy causes overt inflammation in the pleural cavity of TNF KO and TNFR1R2 KO, but not in WT mice (33). Indeed, at day 14 post-infection, the amounts of IFN-γ were 100-fold higher in TNF KO than in WT mice (33). We further explore whether tmTNF controls overt inflammatory environment within the pleural cavity. Following BCG-induced pleurisy, inflammation was confirmed in the pleural cavity of TNF KO mice and was controlled in tmTNF mice that had similar levels of pleural IFN-γ than WT mice (Figure 7A). In contrast to IFN-γ, IFN-α was reduced in TNF KO mice but the levels were similar in tmTNF KI and WT mice (Figure 7B). As the main producers of IFN-γ are CD4 T cells, we analyzed the frequency of CD4 T cells expressing IFN-γ and also IL-17 in the pleural cavity and in the spleen. We found similar results for WT and tmTNF KI cells, but TNF KO had an increased proportion of CD4 T cells expressing IFN-γ and lower frequency of cells producing IL-17 (Figures 7C,D). Splenic CD4 T cells showed only in TNF KO mice an increased frequency IFN-γ producing cells but no differences were observed for cells producing IL-17 (Figures 7E,F). Spleen CD4 T cells were then flow-sorted and activated with BCG (MOI 1) or anti-CD3/anti-CD28. Our results showed that BCG activation of CD4 T cells induced similar amounts of IFN-γ in WT and tmTNF KI cells, while TNF KO cells were over activated producing higher amounts of IFN-γ (Figure 7G). The second activation of CD4 T cells with anti-CD4/anti-CD28 antibodies also enhanced IFN-γ production by tmTNF KI cells compared to WT cells but TNF KO cells produced substantially higher amounts than tmTNF KI cells. This result suggests that TNF KO CD4 T cells are highly responsive to both antigen specific and polyclonal stimuli but tmTNF KI cells have attenuated responses as WT cells (Figure 7H). In conclusion, tmTNF is sufficient for downregulating hyperactivated TNF KO CD4 T cells, thus controlling cell-mediated inflammatory responses.

## Discussion

Tumor necrosis factor is a pleiotropic cytokine pivotal for the development of several human immunopathologies, and also involved in immunoregulatory functions and host defense mechanisms against many pathogens. TNF has been considered a major pro-inflammatory cytokine, however, from accumulating studies it appears that during mycobacterial infection TNF exerts both pro and anti-inflammatory activities that are necessary, first, for a rapid recruitment of cells to infected sites and second to attenuate this process in order to limit tissue injury. *In vitro* and *in vivo* studies suggested that TNF acts as a negative regulator of Th1 immune responses and that TNFR1 signaling is the receptor mediating anti-inflammatory activities during chronic mycobacterial infections (17, 35, 36). However, the underlying mechanisms involved in innate immunity against mycobacteria requiring either tmTNF or solTNF are not elucidated. Our previous studies have shown that the heightened inflammatory reaction during acute *M. tuberculosis* infection caused by the absence of TNF was prevented by the tmTNF form binding to both TNFRs. On the contrary, during chronic infection tmTNF was not sufficient and mice died from overt inflammation and tissue necrosis in spite of the low bacterial burden in infected organs at late stage of infection. This suggests a requirement of solTNF interacting with TNFR1 for disease resolution during chronic infection (18, 19). Our previous study revealed that TNFR1 expressed by myeloid cells, but not by T cells controlled chronic *M. tuberculosis* infection as the absence of TNFR1 on myeloid cells recapitulated the marked impairment in host protection and exacerbated pathology of mice without TNFR1 during *M. tuberculosis* infection (37). Studies assessing the role of TNFR2 during chronic tuberculosis infection have shown that TNFR2 can mediate deleterious effect by soluble TNFR2 shedding inhibiting TNF-associated activities on DC (38). These data suggest that tmTNF interacting with TNFR2 exerts differential activities during acute and chronic infections that depend on several cell types expressing different TNF receptors as well as of the time course of the infection. This study investigates how tmTNF controls the acute inflammatory process generated by BCG-induced pleurisy and reveals that MDSC accumulate in TNF KO mice, but these cells are not functional. However, MDSC expressing tmTNF recover MDSC suppressive function on CD4 T cells, attenuate inflammation limiting tissue injury and rescue tmTNF KI mice. Monocytic MDSC from WT and tmTNF KI mice inhibited CD4 T cells proliferation in association with inhibition of IL-2, IFN-γ and iNOS production. Granulocytic MDSC also inhibited CD4 T cells, however, the cytokine profile was not as clearly defined as for MO-MDSC, but iNOS was also expressed at much lower levels in TNF KO cells. We then examine the specific receptor sustaining MDSC function. We find that proliferation of activated CD4 T cells deprived of TNFR2 were not inhibited by MDSC, suggesting that the interaction of tmTNF expressed by MDSC and TNFR2 on CD4 T cells is critical for MDSC-mediated T cell suppression. It is important to note that absence of TNFR2 on T cells led to contrary effects as CD4 T cells display enhanced proliferation and enhanced IFN-γ production when co-cultured with MDSC. The proliferation capacity of TNFR2-deficient CD4 T cells was lower than that of WT cells as previously reported (39) which was shown to be normal in other report (40). Our data show that the proliferation CD4 T cells deficient in TNFR2 was not influenced by the presence of either WT or tmTNF KI MDSC. By contrast, the proliferation CD4 T cells deficient in TNFR1 was inhibited by both WT and tmTNF KI MDSC, suggesting the importance of TNFR2 on CD4 T cell suppressive activity. Thus, MDSC expressing tmTNF appears to control BCG-induced pleurisy *via* TNFR2 on CD4 T cells. Expression of tmTNF on MDSC has not been explored so far. To our knowledge, we describe here for the first time that tmTNF expressed by MDSC exerts suppressive activity on T cells expressing TNFR2 during acute BCG-induced pleurisy.

The role of TNF has been shown to be critical for the generation of MDSC during several pathologies, including cancer and chronic inflammation (24, 26, 41). Suppressive function of MDSC on T cells was shown to be dependent on the presence of TNFR2 on MDSC which could help tumor cells to evade the immune system (26). Ectopic expression of tmTNF on tumor cells promoted suppressive activity of MDSC expressing TNFR2 (41). In mouse models of carcinogenesis, neutralization of TNF by etanercept and infliximab resulted in reduced MDSC accumulation and delayed growth of transplanted tumors (27). Inhibition of solTNF by dominant-negative TNF biologics, blocking solTNF but not tmTNF, decreased MDSC frequency, reduced tumor growth, and prolonged survival of mice with chemically induced tumors, suggesting that solTNF was responsible for MDSC accumulation during carcinogenesis (42). TNF has been shown to act as a pro and anti-tumorigenic molecule depending on the different phases of carcinogenesis (43). Lymphotoxin-alpha (LT-α) also signaling through TNFR1 and TNFR2 can also contribute and impact on MDSC accumulation and expansion which indicates that TNF/LT-α pathways are major and complex targets in carcinogenesis.

Tumor necrosis factor signaling through TNFR2 has been shown to be required for MDSC accumulation and suppressive activities as also reported for T regulatory cells (25, 26, 44–46). Our data confirm that this important pathway tmTNF-TNFR2 preferentially leads to the activation of tolerogenic MDSC that are involved in anti-inflammation and infection resolution. TNFR2 expressed by T cells has been reported to act as a co-stimulatory molecule for antigen-driven T cell responses (39). TNF has been shown to activate suppression activity of regulatory T cells (Tregs) by inducing the expression of TNFR2 (47). BCG vaccination has been shown to activate Tregs mainly in the context of auto-immunity and diabetes (48). The exploration of Tregs functionality in the context of BCG pleural infection needs further investigation. Nevertheless, we found a very low proportion of Tregs in the pleural cavity of infected mice (0.3–0.4%) (data not shown) and very low levels of IL-10 (20–50 pg/mL), indicating a relative contribution of Tregs at this time point of the infection.

Several reports have explored the role of granulocytic and monocytic MDSC during chronic mycobacterial infections in the mouse model and in TB patients; however, results in terms of T cell responses are not totally clear probably due to the different model systems (11, 13, 14). During chronic murine TB, MDSC accumulation in the lung was increased in susceptible mice and associated with heightened lethality, but depletion of MDSC during infection ameliorated disease (15). In patients with TB, MDSC accumulation was identified in the blood and after successful treatment the frequency of MDSC was decreased as seen in healthy controls (9). Pulmonary accumulation of granulocytic MDSC expressing NO was also reported in TB patients (10). In general, studies on MDSC in TB showed that MDSC may contribute to the pathogenesis of TB, and in particular in susceptible hosts, MDSC were associated with disease aggravation (16, 49). However, studies on the role of MDSC during the initial phase of the infection are still missing. As in the case for TNF requirement that needs to be at the right time with sufficient levels to be efficient, MDSC can also exert protective activity during a specific time of the infection to prevent overt inflammation whereas they can be deleterious during chronicity. Contrary to previous results performed during chronic phase infection, our study on acute infection suggests that MDSC play a beneficial role by attenuating T-cell-mediated inflammatory responses.

**Figure 8:**
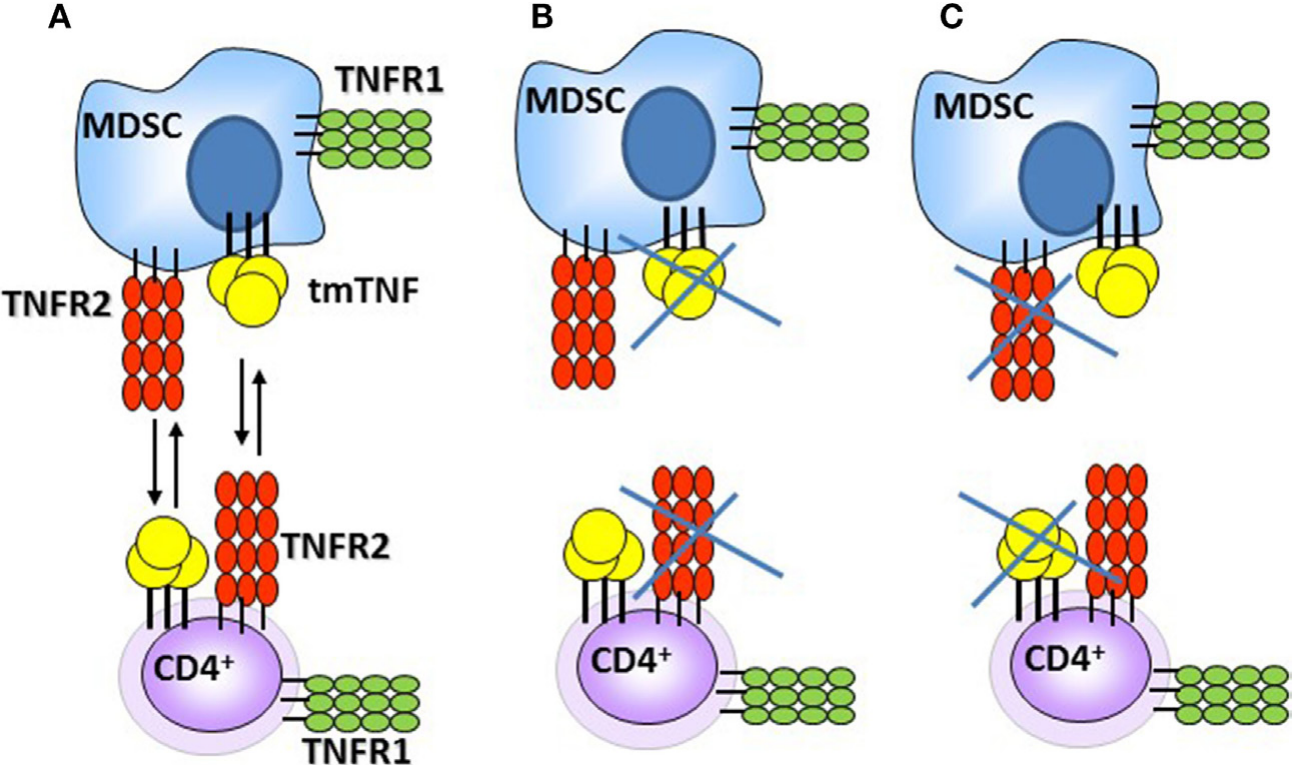
Schematic representation of the interactions between myeloid-derived suppressor cells (MDSC) and CD4 T cells during acute BCG pleural infection. **(A)** Interactions triggering suppressive effects of MDSC on CD4 T cells *via* tmTNF and TNFR2. **(B,C)** Absence of tmTNF or TNFR2 abrogates MDSC suppressive activities [presented data and Ref. (25, 26, 41)].

Previous studies have shown that TNF acts as a negative regulator of Th1 immune responses as in the absence of TNF expansion of T cells and uncontrolled Th1 type immune responses caused tissue destruction (35). Our previous report also pointed out that the functional tmTNF^Δ1–9,K11E^ controlled the exacerbated serum IFN-γ levels observed in TNF KO. By contrast, a second mouse strain (tmTNF^Δ1–12^ KI mice) expressing a different mutant tmTNF^Δ1–12^ were unable to control the BCG infection and exhibited high IFN-γ levels associated with aggravation of the disease and death as TNF KO mice (31). In this study, BCG-infected TNF KO mice exhibited excessive levels of IFN-γ, as previously observed, and impaired response in IFN-α in the pleural cavity. We show that expression of tmTNF^Δ1–9,K11E^ regulated both IFN-γ and IFN-α with attenuation of the Th1 cell-mediated inflammatory responses in the pleural cavity of BCG-infected mice. This anti-inflammatory effect would result from the interaction of tmTNF on MDSC with TNFR2 on CD4 T cells. We also examined whether TNF signaling was needed for pleural MDSC suppressive activity and showed that TNF signaling on MDSC is important for CD4 T cell suppressive function during acute pleural BCG infection. TNFR1R2 KO MDSC trigger a contrary effect enhancing CD4 T cell proliferation and production of IFN-γ which recapitulates the effects observed with co-cultures of CD4 T cells deficient in TNFR2. These results suggest that MDSC requires the presence of tmTNF and also of TNFRs, most probably TNFR2 as previously reported (25, 26, 41). Based on our data and previous report, we propose that MDSC–CD4 T cell interactions can be mediated through tmTNF-TNFR2 and cells can express both tmTNF and TNFRs (Figure 8). Interaction of tmTNF with TNFR1 or TNFR2 can result in the transmission of different signals, including reverse signaling which remains to be investigated in MDSC – T cell interactions (50).

In conclusion, our study provides insights into the protective role of MDSC during acute mycobacterial infection that involves tmTNF signaling through TNFR2. Tm-TNFR2 interaction attenuates cell-mediated inflammatory responses associated with the infection and favors adaptive immunity and disease resolution.

## Ethics Statement

This study was approved by Cantonal veterinary office from Geneva (authorization No. GE167/14).

## Author Contributions

Conception and drafting of the article: LC-G and IG. Performance and analysis of experiments: LC-G, IG, HU, DV, and GB. MB for discussions of the data and critical revision of the article: VQ and BR.

## Conflict of Interest Statement

The authors declare that the research was conducted in the absence of any commercial or financial relationships that could be construed as a potential conflict of interest.

## Supplementary Material

Figure S1 Cytokine profile of pleural fluid WT and tmTNF KI mice. **(A)** IFN-γ, **(B)** IL-12p70, **(C)** IL-6, and **(D)** IL-10 cytokine levels and **(E)** the chemokine MCP-1 (CCL2) were evaluated in the pleural fluid of naive mice and after 2 and 14 weeks of BCG-induced pleurisy [bar graphs show means ± SEM, *n* = 6–14/per group, **P* < 0.05 vs WT, ANOVA and Bonferroni *post hoc* test from two experiments].

Figure S2 Transmembrane TNF controls CD11^+^ GR1^+^ cell expansion in the pleural cavity of BCG-infected mice. **(A)** Representative zebra plot showing pleural cells expressing CD11b and GR1 from naïve mice and BCG-infected mice at day 14 post-infection. **(B)** Absolute number of pleural CD11b^+^ GR1^+^ cells obtained from total cell number recovered from pleural cavity per individual mouse [bar graphs are means ± SEM, *n* = 3–5 naïve condition and *n* = 6–8 for infected mice/per group from 2 experiments, **P* < 0.05 vs WT. ANOVA and Bonferroni *post hoc* test].

Figure S3 Absence of TNF induces low frequency of MDSC with ability to produce iNOS and Arginase 1 in the pleural cavity of BCG-infected mice, even using CD11b and GR1 as main markers. MDSC were flow-sorted from total pleural cells using a MDSC kit. **(A)** Representative zebra plot with the analysis used to evaluate the purity of PMN-MDSC by flow cytometry, using CD11b and GR1 to identify MDSC cells. **(B)** Western blot of flow-sorted PMN-MDSC showing expression of iNOS and arginase-1 (Arg 1) in WT and tmTNF KI cells but less in TNF KO cells. **(C)** Representative Stagger Offset histogram showing the proportion of PMN-MDSC expressing iNOS inside the gate of CD11b^+^ GR1^+^ cells and comparison between WT (blue), TNF KO (orange) and tmTNF KI (green) mice. **(D)** Histogram representing western blot quantification compared to β-actin **(E)** Representative zebra plot with the analysis used to evaluate the purity of MO-MDSC by flow cytometry, using as main molecules CD11b and GR1 to identify MDSC. **(F)** Western blot of flow-sorted MO-MDSC showing expression of iNOS and Arg 1 in WT and tmTNF KI cells but not in TNF KO cells. Beta actin was used as control and TNF KO cells are over loaded. **(G)** Representative Stagger Offset histogram showing the proportion of MO-MDSC expressing iNOS inside the gate CD11b^+^ GR1 (left) and comparison between WT (blue), TNF KO (orange), and tmTNF KI (green) mice. **(H)** Histogram representing western blot quantification compared to β-actin.

Figure S4 Gating strategy for evaluation of CD4 T cell proliferation. Flow cytometry analysis to evaluate CD4 T cell proliferation following activation with anti CD3 1 µg/mL (Plate-immobilized) plus anti CD28 1 µg/mL and after 48 h of culture and using KI-67 proliferation marker.

Figure S5 Expression of TNFRs on MDSC is required MDSC suppressive function on CD4 T cells. **(A)**. Proliferation of CD3 CD4 T cells after polyclonal stimulation and in the presence or absence of flow-sorted pleural mononuclear MO-MDSC (ratio MDSC:Splenocytes, 1:1, 1:2, and1:4) was measured by flow cytometry using KI-67 after 48 h of co-culture. Pools of pleural cells were from 5 to 7 mice per group. Sorted MDSC were from WT BCG-infected mice or from TNFR1TNFR2 KO mice. **(B)** IL-2 and **(C)** IFN-γ production from supernatants of splenocytes and MO-MDSC co-cultures at different ratio. **(D)** Proliferation of CD3 CD4 T cells after polyclonal stimulation and in the presence or absence of flow-sorted pleural polymorphonuclear PMN-MDSC co-cultured with splenocytes for 48 h. **(E)** IL-2 and **(F)** IFN-γ production from co-cultures of PMN-MDSC and splenocytes. MDSC alone were used as the negative control and activated splenocytes as positive controls (100%). Bar graphs show means ± SEM. Data are representative of two independent experiments (**P* < 0.05 vs positive control. ANOVA and Bonferroni *post hoc* test).
