# Supplementary material for: Transmembrane Tumor Necrosis Factor Controls Myeloid-Derived Suppressor Cell Activity via TNF Receptor 2 and Protects from Excessive Inflammation during BCG-Induced Pleurisy
Source: Front Immunol. 2017 Aug 25;8:999. doi: 10.3389/fimmu.2017.00999 (PMC5574880; doi:10.3389/fimmu.2017.00999)
Supplement: Figure S1 — Cytokine profile of pleural fluid WT and tmTNF KI mice. (A) IFN-γ, (B) IL-12p70, (C) IL-6, and (D) IL-10 cytokine levels and (E) the chemokine MCP-1 (CCL2) were evaluated in the pleural fluid of naive mice and after 2 and 14 weeks of BCG-induced pleurisy [bar graphs show means ± SEM, n = 6–14/per group, *P < 0.05 vs WT, ANOVA and Bonferroni post hoc test from two experiments]. [file Data_Sheet_1.PDF]

SUPPLEMENTARY FIGURES

S1 Fig. Cytokine profile of pleural fluid WT and tmTNF KI mice

(A) IFN-g, (B) IL-12p70, (C) IL-6, and (D) IL-10 cytokine levels and (E) the chemokine MCP-1 (CCL2) were evaluated in the pleural fluid of naive mice and after 2 and 14 weeks of BCG-induced pleurisy (bar graphs show means ± SEM (n=6-14/ per group, *p<0.05 vs WT, ANOVA and Bonferroni post-hoc test from 2 experiments).

S2 Fig. Transmembrane TNF controls CD11+GR1+ cell expansion in the pleural cavity of BCG–infected mice.

(A) Representative zebra plot showing pleural cells expressing CD11b and GR1 from naïve mice and BCG-infected mice at day 14 post-infection. (B) Absolute number of pleural CD11b+GR1+ cells obtained from total cell number recovered from pleural cavity per individual mouse (bar graphs are means ± SEM (n= 3 to 5 naïve condition and n= 6 to 8 for infected mice/ per group from 2 experiments. *P<0.05 vs WT. ANOVA and Bonferroni post-hoc test).

S3 Fig. Absence of TNF induces low frequency of MDSC with ability to produce iNOS and Arginase 1 in the pleural cavity of BCG-infected mice, even using CD11b and GR1 as main markers.

MDSC were flow-sorted from total pleural cells using a MDSC kit. (A) Representative zebra plot with the analysis used to evaluate the purity of PMN-MDSC by flow cytometry, using as main molecules CD11b and GR1 to identify MDSC cells. (B) Western blot of flow-sorted PMN-MDSC showing expression of iNOS and arginase-1 (Arg 1) in WT and tmTNF KI cells but less in TNF KO cells.

(C) Representative Stagger Offset histogram showing the proportion of PMN-MDSC expressing iNOS inside the gate of CD11b+GR1+ cells and comparison between WT (blue), TNF KO (orange) and tmTNF KI (green) mice. (D) Histogram representing western blot quantification compared to β-actin (E) Representative zebra plot with the analysis used to evaluate the purity of MO-MDSC by flow cytometry, using as main molecules CD11b and GR1 to identify MDSC. (F) Western blot of flow-sorted MO-MDSC showing expression of iNOS and arginase-1 in WT and tmTNF KI cells but not in TNF KO cells. Beta actin was used as control and TNF KO cells are over loaded. (G) Representative Stagger Offset histogram showing the proportion of MO-MDSC expressing iNOS inside the gate CD11b+ GR1 (left) and comparison between WT (blue), TNF KO (orange) and tmTNF KI (green) mice. (H) Histogram representing western blot quantification compared to β-actin.

S4 Fig. Gating strategy for evaluation of CD4 proliferation.

Flow cytometry analysis to evaluate CD4 T cell proliferation following activation with anti-CD3 (1 µg/mL) (Plate-immobilized) plus anti CD28 1µg/mL and after 48h of culture and using KI-67 proliferation marker.

S5 Fig. Expression of TNFRs on MDSC is required MDSC suppressive function on CD4 T cells.

(A). Proliferation of CD3 CD4 T cells after polyclonal stimulation and in the presence or absence of flow-sorted pleural mononuclear MO-MDSC (ratio MDSC:Splenocytes, 1:1, 1:2 and 1:4) was measured by flow cytometry using KI-67 after 48 hrs of co-culture. Pools of pleural cells were from 5-7 mice per group. Sorted MDSC were from WT BCG infected mice or from TNFR1TNFR2 KO mice. (B) IL-2 and (C) IFN-γ production from supernatants of splenocytes and MO-MDSC co-cultures at different ratio. (D) Proliferation of CD3 CD4 T cells after polyclonal stimulation and in the presence or absence of flow-sorted pleural polymorphonuclear PMN-MDSC co-cultured with splenocytes for 48 hrs. (E) IL-2 and (F) IFN-γ production from co-cultures of PMN-MDSC and splenocytes. MDSC alone were used as the negative control and activated splenocytes as positive controls (100%). Bar graphs show means ± SEM. Data are representative of 2 independent experiments (n=3 to 6 per group, *P<0.05 versus positive control. ANOVA and Bonferroni post-hoc test).

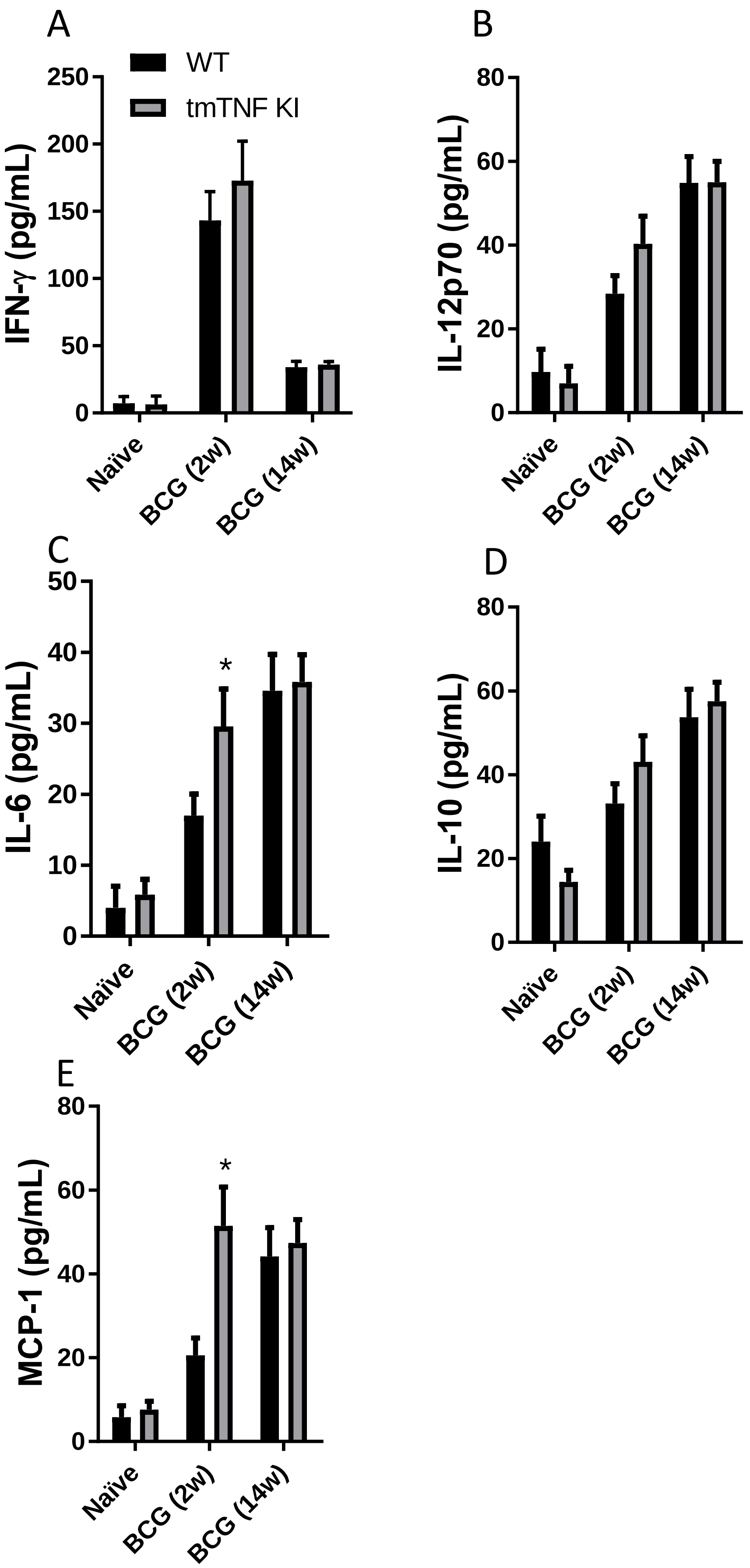
A
WT
tmTNF KI
IFN-γ (pg/mL)
250
200
150
100
50
0
Naïve
BCG (2w)
BCG (14w)
B
IL-12p70 (pg/mL)
80
60
40
20
0
C
IL-6 (pg/mL)
50
40
30
20
10
0
*
D
IL-10 (pg/mL)
E
MCP-1 (pg/mL)
*

S1 Fig

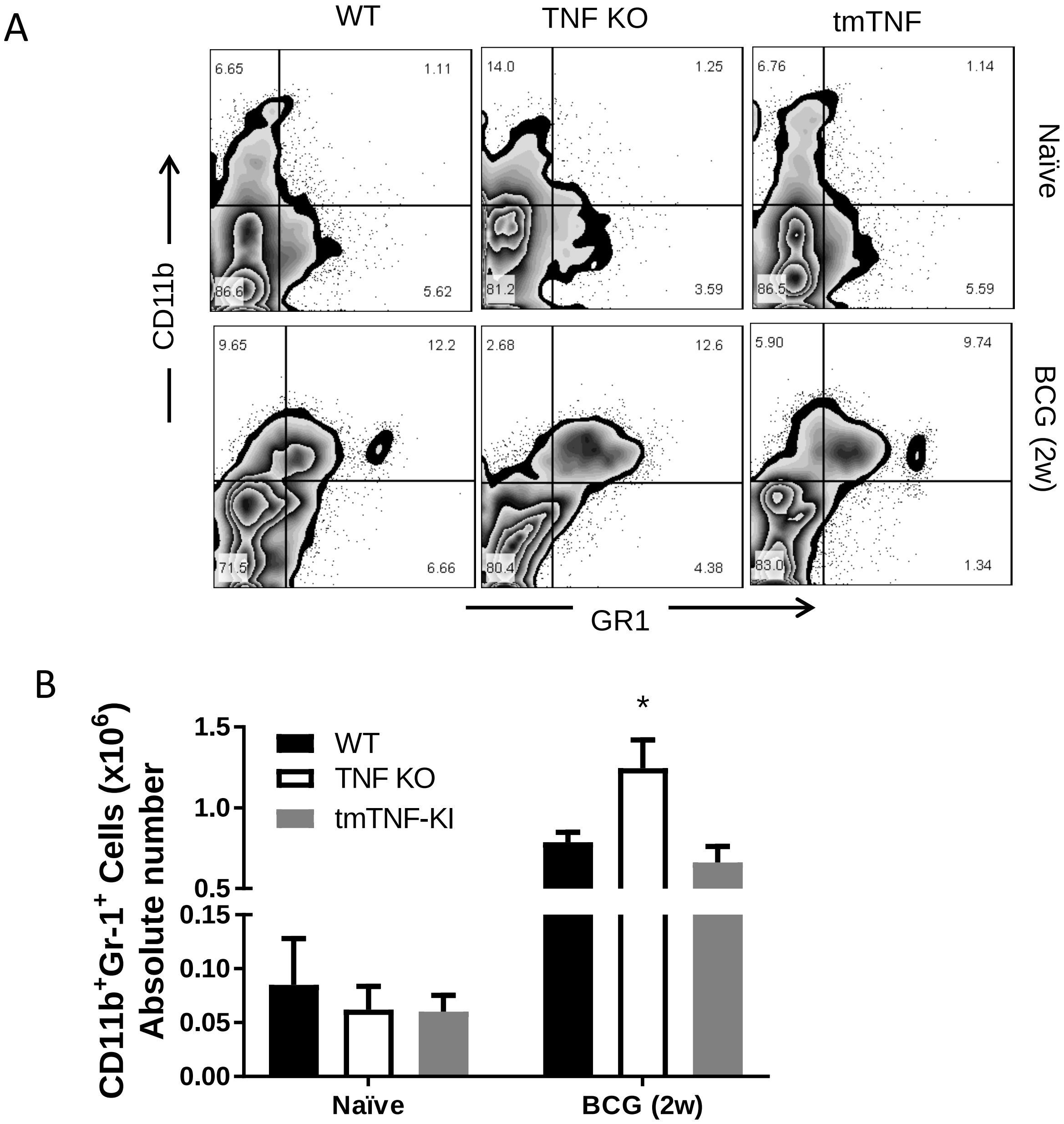
A
WT
TNF KO
tmTNF
CD11b
GR1
Naïve
BCG (2w)
6.65
1.11
86.6
5.62
14.0
1.25
81.2
3.59
6.76
1.14
86.5
5.59
9.65
12.2
71.5
6.66
2.68
12.6
80.4
4.38
5.90
9.74
83.0
1.34
B
CD11b+Gr-1+ Cells (x10^6)
Absolute number
WT
TNF KO
tmTNF-KI
*
1.5
1.0
0.5
0.15
0.10
0.05
0.00
Naïve
BCG (2w)

S2 Fig

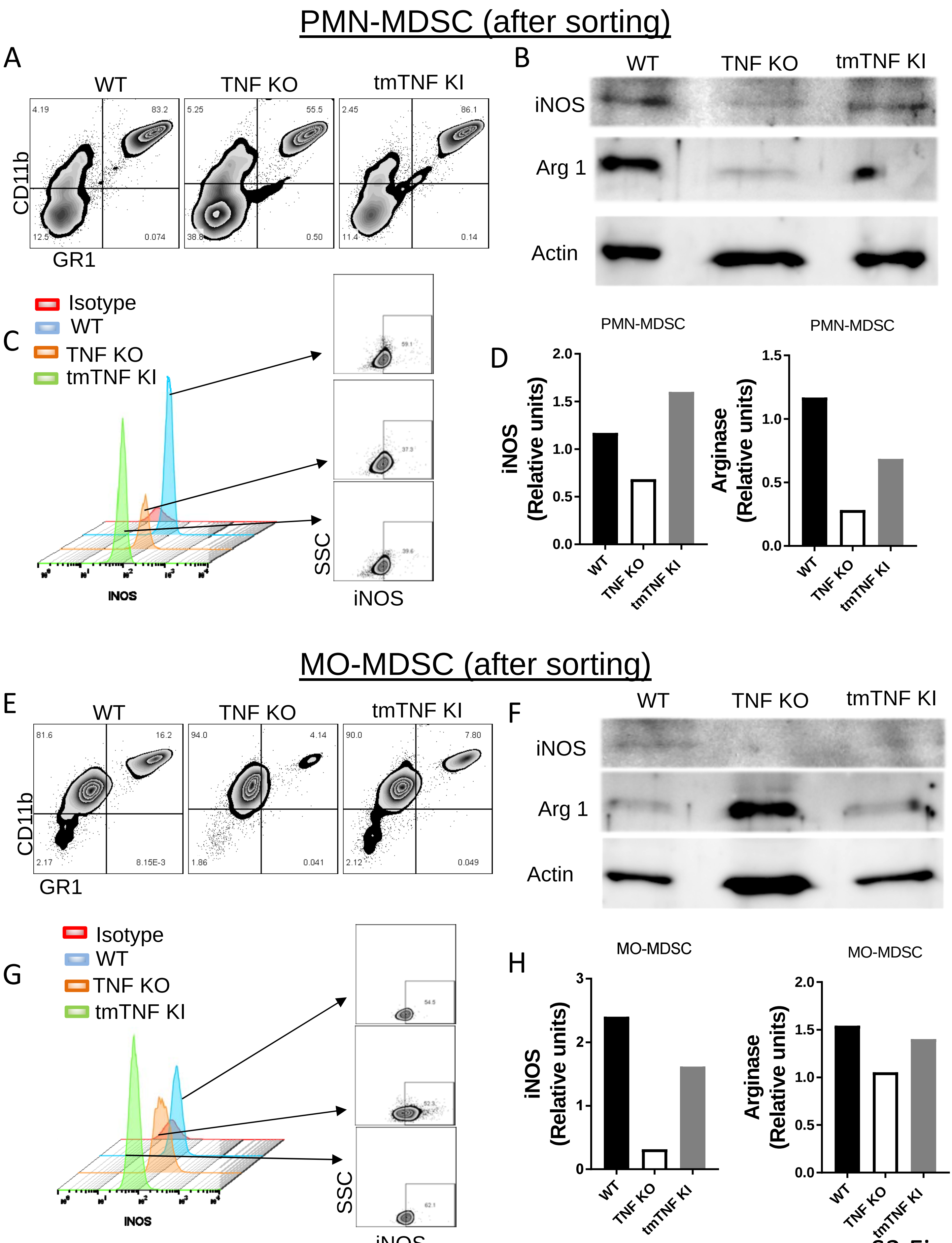
PMN-MDSC (after sorting)
A
WT
TNF KO
tmTNF KI
CD11b
GR1
4.19
83.2
12.5
0.074
5.25
55.5
38.8
0.50
2.45
86.1
11.4
0.14
B
WT
TNF KO
tmTNF KI
iNOS
Arg 1
Actin
C
Isotype
WT
TNF KO
tmTNF KI
iNOS
SSC
iNOS
D
PMN-MDSC
iNOS
(Relative units)
PMN-MDSC
Arginase
(Relative units)
WT
TNF KO
tmTNF KI
MO-MDSC (after sorting)
E
WT
TNF KO
tmTNF KI
CD11b
GR1
81.6
16.2
2.17
8.15E-3
94.0
4.14
1.86
0.041
90.0
7.80
2.12
0.049
F
WT
TNF KO
tmTNF KI
iNOS
Arg 1
Actin
G
Isotype
WT
TNF KO
tmTNF KI
iNOS
SSC
iNOS
H
MO-MDSC
iNOS
(Relative units)
MO-MDSC
Arginase
(Relative units)
WT
TNF KO
tmTNF KI

S3 Fig

Strategy for the analysis of proliferation

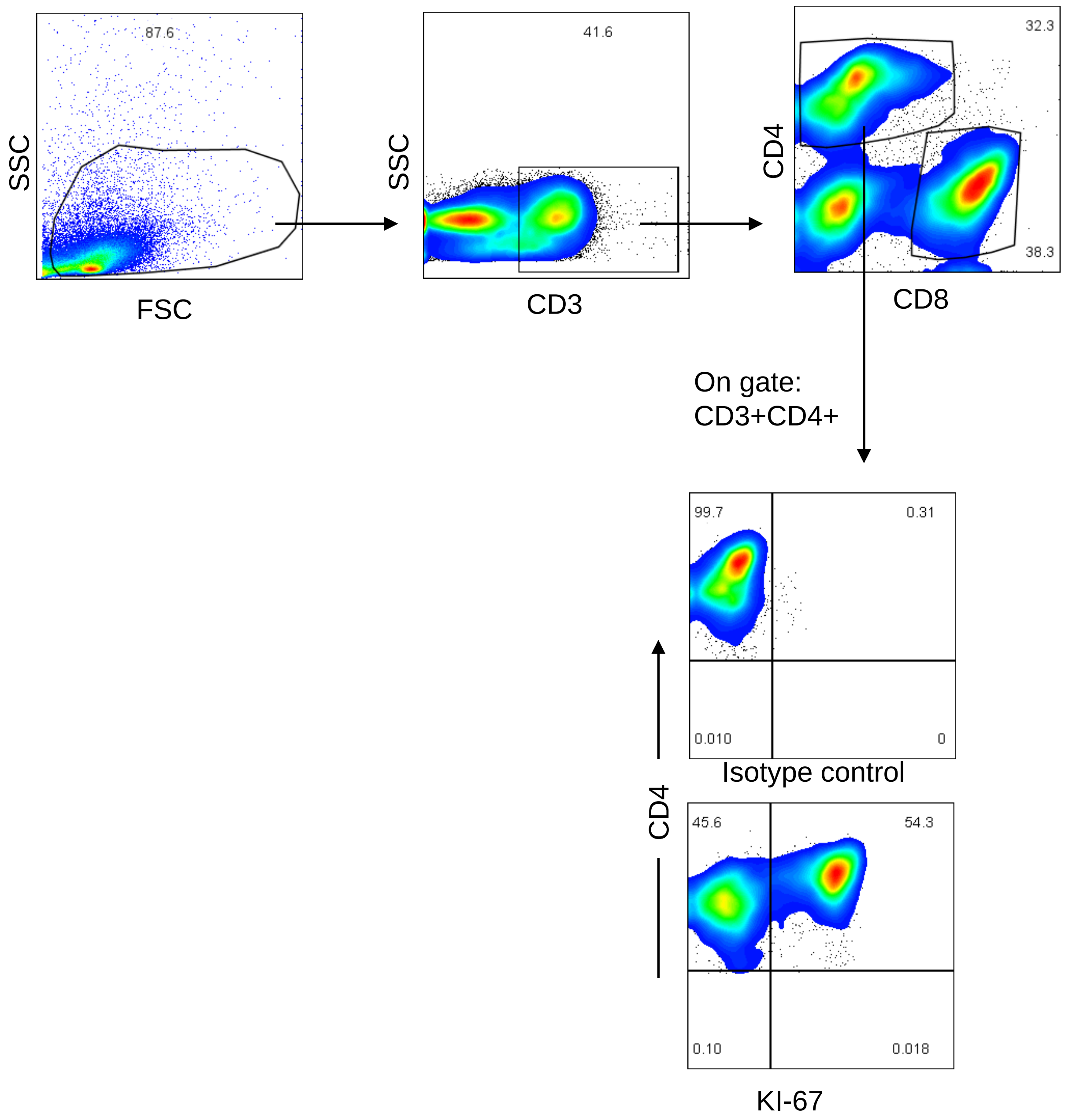

S4 Fig

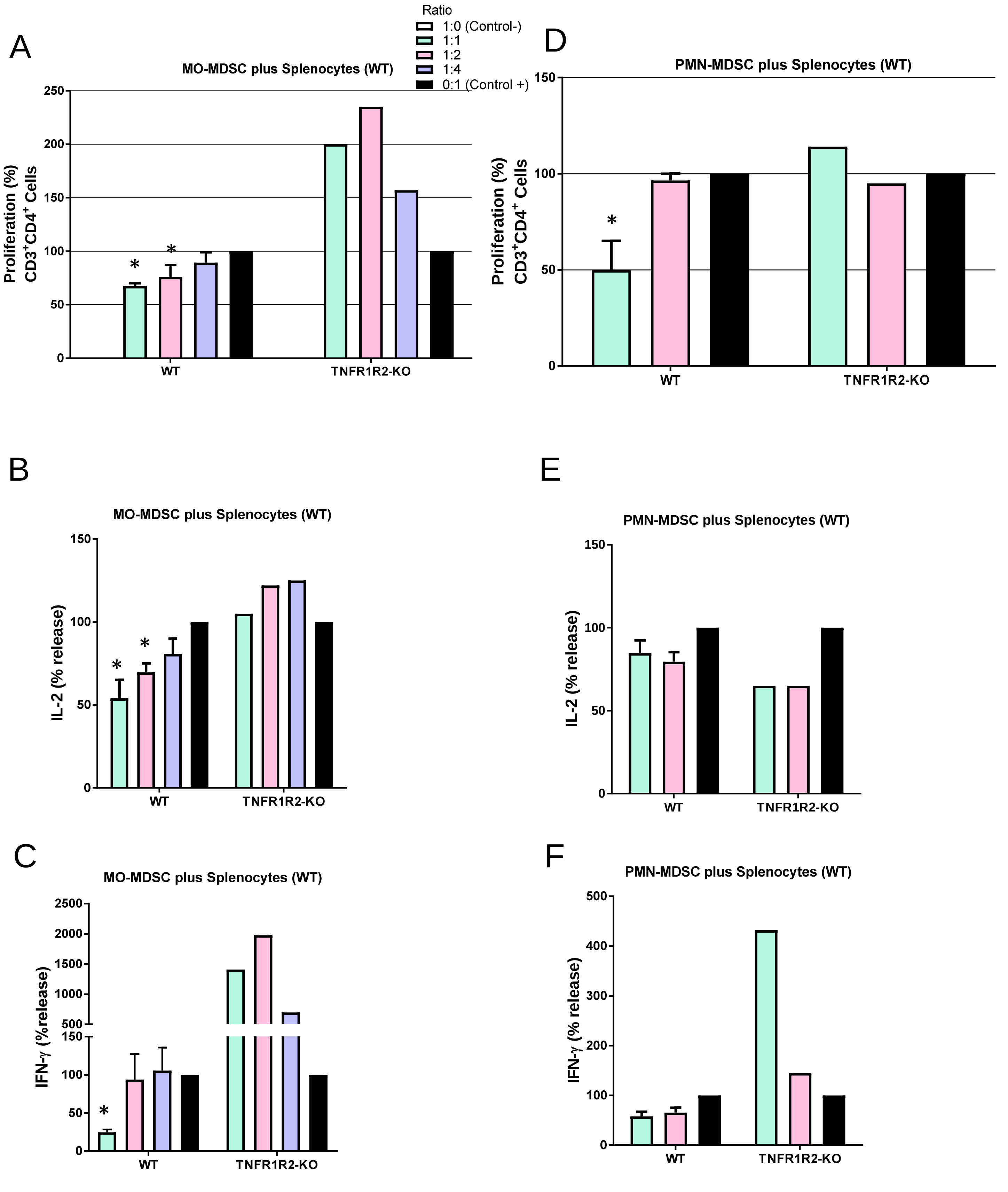
A
MO-MDSC plus Splenocytes (WT)
Ratio
1:0 (Control-)
1:1
1:2
1:4
0:1 (Control +)
Proliferation (%) CD3+CD4+ Cells
250
200
150
100
50
0
*
*
WT
TNFR1R2-KO
D
PMN-MDSC plus Splenocytes (WT)
150
100
50
0
*
WT
TNFR1R2-KO
B
MO-MDSC plus Splenocytes (WT)
IL-2 (% release)
150
100
50
0
*
*
WT
TNFR1R2-KO
E
PMN-MDSC plus Splenocytes (WT)
IL-2 (% release)
150
100
50
0
WT
TNFR1R2-KO
C
MO-MDSC plus Splenocytes (WT)
IFN-γ (%release)
2500
2000
1500
1000
500
150
100
50
0
*
WT
TNFR1R2-KO
F
PMN-MDSC plus Splenocytes (WT)
IFN-γ (% release)
500
400
300
200
100
0
WT
TNFR1R2-KO

S5 Fig
